# Congenital Anterior Urethrocutaneous Fistula with a Persistent Urethral Groove

**DOI:** 10.1055/s-0040-1721469

**Published:** 2021-01-29

**Authors:** Hazem Mosa, Massimo Garriboli

**Affiliations:** 1 Department of Paediatric Urology, Evelina London Children's Hospital, London, United Kingdom

**Keywords:** congenital anterior urethrocutaneous fistula, CAUF, hypospadias

## Abstract

Congenital anterior urethrocutaneous fistula (CAUF) is a rare penile anomaly with only 63 cases reported in the literature. The anomaly can present in isolation or in association with chordee or hypospadias. We report the case of an 8-month-old boy with CAUF that resembles the embryological urethral groove. On examination, a wide urethral groove was noted to cover the midshaft of the penis with a well formed urethra extending proximally and distally and with a normal glandular anatomy, a wide glandular meatus, and a complete foreskin. The urethral groove was tubularized and covered in layers. Surgery was complicated with early superficial skin dehiscence not affecting the urethral repair.

Refashioning of the skin was then performed. A satisfactory aesthetic and functional outcome was observed at 7 years' follow-up. Defining the anatomy of CAUF and distal urethra is key in management of these children.

**New Insights and the Importance for the Pediatric Surgeon**

Understanding the anatomy and embryology of congenital anterior urethrocutaneous fistula is key in management of these children. This is essential for making the correct decision of which surgical repair to perform.

## Introduction

Urethrocutaneous fistula and its surgical treatment is one of the most discussed topics in the context of complications of hypospadias surgery 1 ; congenital anterior urethrocutaneous fistula (CAUF) is very rare with only 63 cases reported in the literature. 2 Authors have described two distinct anatomical variants; one associated with a hypoplastic distal urethra, chordee, and a hooded foreskin, this variant has also been reported in association with anorectal malformation, 2 and the other being an isolated congenital fistula with normal foreskin, normal glans fusion, and normal urethral development proximal and distal to the fistula. There is some confusion about terminology used in the literature to describe these cases with some authors describing them as Y urethral duplication variants. 2 The operative management is directed by the anatomical features of each patient. We describe the case of a boy who presented with a CAUF characterized by a wide mucosal plate that looked similar to the embryological urethral groove.

## Case Report

**Fig. 1:**
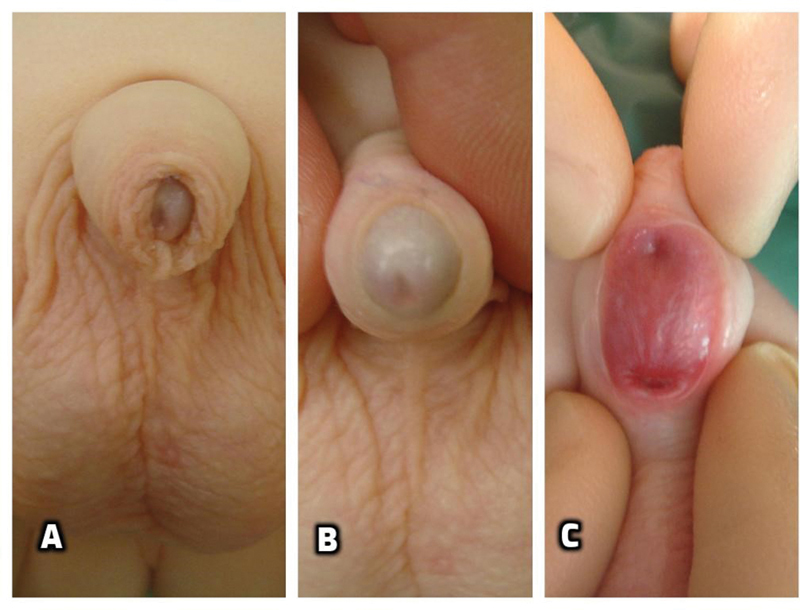
Congenital anterior urethrocutaneous fistula (CAUF) with a large fistula: preoperative examination showed complete foreskin ( **A** ), normal glans with a wide orthotopic meatus ( **B** ), and a persistent urethral groove with two openings communicating to distal (arrow head) and proximal (arrow) urethra ( **C** ).

An 8-month-old boy was referred to our pediatric urology department with concerns related to the anatomy of his penis. No history of previous penile surgery or trauma was reported. On examination, the glans looked well formed with a normal pediatric foreskin and a normal, wide glandular meatus. A large defect within the midshaft was present. The defect measured 3 × 2 cm and was lined with mucosa of the urethral groove similar to the lining of the urethral plate in patients with hypospadias. Two openings were seen which communicated to the distal and proximal urethra ( Fig. 1 ). The patient underwent an examination under general anesthesia that revealed a wide patent distal urethra well covered with spongiosum; the proximal urethra was also normal on cystoscopic examination and did not demonstrate any obstruction nor the presence of any accessory, duplicated urethra. At the age of 11 months, the urethral groove was closed primarily using the Thiersch‒Duplay technique with a continuous 7–0 synthetic absorbable monofilament suture. A second waterproof layer was added using local ventral dartos tissue. The skin was then closed in two layers using 6–0 synthetic absorbable monofilament interrupted sutures. A 6 F feeding tube was used for stenting the repair. Dressing and catheter were removed 7 days postoperatively as per our usual protocol. The patient developed an early superficial skin dehiscence without signs of infection or inflammation. This was managed operatively by excising the margins, mobilizing the skin laterally, and closing it over the tubularized urethra in three layers. At the time of the second procedure, the urethral repair was found intact and did not require intervention. After the second repair a small area of the skin had a further minor dehiscence and was allowed to heal by second intention. From the functional point of view the boy had a successful repair and always demonstrated normal urinary stream. After follow-up of 7 years, the boy is fully continent and voids normally.

## Discussion

CAUF is an extremely rare condition. The largest case series (14 cases) has been reported in 1999 by Caldamone et al. 3 Lin et al, in their systematic review, reported 63 cases between 1962 and 2017. 2 The most common fistula location was subcoronal, followed by the mid-penile with proximal penile and penoscrotal being the least reported location. 4

Olbourne theorized that a defective urethral plate folding might explain mid-penile congenital fistulae as a consequence of partially disrupted urethral fold fusion. 5 The extent of disruption of the folding process of the urethral groove may determine the size of the fistula. The anatomy of the case we describe differs from those previously published because it presented with a large gap between proximal and distal urethra, rather than a focal defect along the urethra, and because of the presence of a wide urethral groove that suggests a complete failure of the process of tubularization ( Fig. 1C ).

**Fig. 2:**
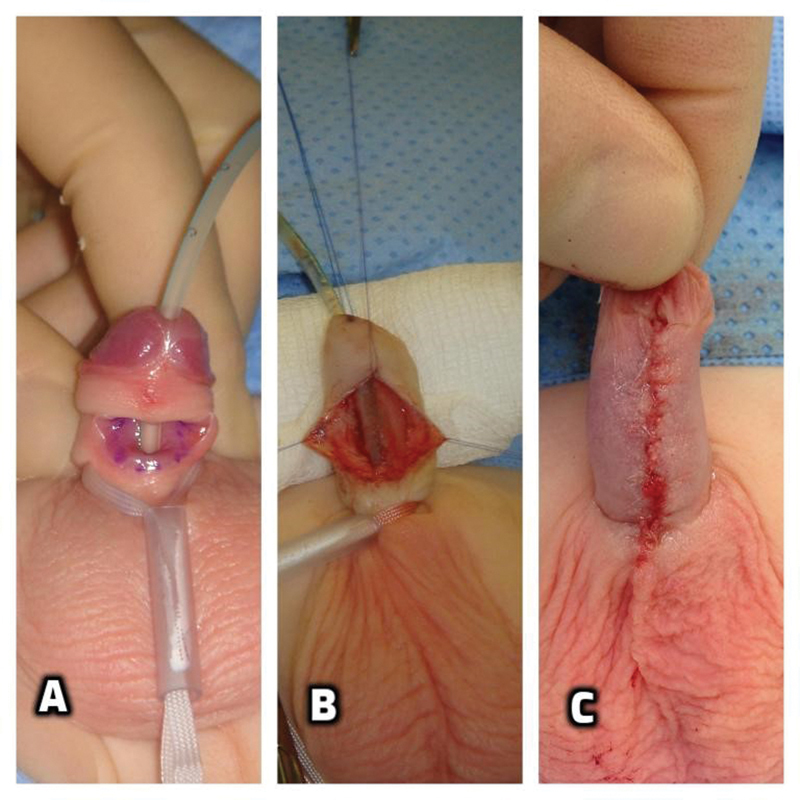
Intraoperatively, a 6 F catheter was passed through normal caliber distal and proximal urethra ( **A** ). Thiersch‒Duplay continuous urethroplasty ( **B** ). Skin closure and final intraoperative appearance ( **C** ).

Two distinct anatomical variants of CAUF have been described by various authors. The first variant has a hypoplastic urethra distal to the fistula and could also be considered a variant of proximal hypospadias. The second variant describes normal urethral development distal and proximal to the fistula as demonstrated in our patient. We agree with Maarafie and Azmy that the distinction between the two variants is essential for planning operative repair. 6 Primary closure of the fistula in layers can be successfully performed for a discrete fistula. On the contrary, a hypoplastic distal urethra requires more extensive dissection with excision of the thin urethra essentially turning it into a distal hypospadias and formally repairing it with urethroplasty and glansplasty. Our case could be considered in between the two variants. To correct it, we performed a Thiersch‒Duplay tubularization of the urethral groove while preserving a normally formed glans and distal urethra ( Fig. 2B ).

Hassan et al recommended a preoperative cystourethrogram for differentiating a congenital fistula from a urethral duplication. 7 8 This may be of value in small fistulae while in our case we confirmed the presence of normal and patent urethra both distally and proximally during an examination under general anesthesia and cystoscopy. If a Y duplication of the urethra is suspected, a preoperative micturating cystourethrogram can help to define the anatomy. Effmann's type 2A duplications can present as discrete fistulae along the penile shaft with a normal orthotopic meatus. 9 Other associated genitourinary abnormalities have been reported and include duplex kidneys, solitary kidneys, megalourethra, and vesicoureteral reflux. 2

In the published cases, postoperative complications are described in approximately 11% of the patients, mostly being recurrence of a fistula. 2 Another interesting element that characterizes our case is the fact that, while the urethroplasty was successful and despite a tension-free closure, an early skin dehiscence has occurred. This occurred without evidence of trauma, inflammation, or infection which lead us to wonder if factors relating to wound healing are disrupted in the skin adjacent to the persistent urethral groove.

## Conclusion

We present a case of CAUF with an unusually large fistula with mucosal lining resembling the embryological urethral groove.

Defining the anatomy of CAUF and distal urethra is key in managing these children. However, the complex and variable anatomy and embryology of CAUF are not yet fully understood.
